# Early onset fetal growth restriction

**DOI:** 10.1186/s40748-016-0041-x

**Published:** 2017-01-18

**Authors:** Andrea Dall’Asta, Valentina Brunelli, Federico Prefumo, Tiziana Frusca, Christoph C Lees

**Affiliations:** 1 Centre for Fetal Care, Queen Charlotte’s and Chelsea Hospital, Imperial College Healthcare NHS Trust, Du Cane Road, London, W12 0HS United Kingdom; 2 Department of Obstetrics & Gynecology, University of Parma, Parma, Italy; 3 Department of Obstetrics and Gynaecology, Maternal-Fetal Medicine Unit, University of Brescia, Brescia, Italy; 4 Department of Surgery and Cancer, Imperial College London, London, United Kingdom; 5 Department of Development and Regeneration, KU Leuven, Belgium

**Keywords:** Fetal Doppler, Fetal complications, Iatrogenic preterm delivery, Antenatal counseling, Neonatal intensive care unit

## Abstract

Fetal growth restriction (FGR) diagnosed before 32 weeks is identified by fetal smallness associated with Doppler abnormalities and is associated with significant perinatal morbidity and mortality and maternal complications. Recent studies have provided new insights into pathophysiology, management options and postnatal outcomes of FGR. In this paper we review the available evidence regarding diagnosis, management and prognosis of fetuses diagnosed with FGR before 32 weeks of gestation.

## Background

Fetal growth restriction (FGR) is both a common obstetric condition and a major cause of perinatal morbidity and mortality [1, 2]. Early FGR by definition is diagnosed at or below 32 weeks and differs from late onset FGR also in terms of its clinical manifestations, association with hypertension [3], patterns of deterioration and severity of placental dysfunction [4, 5].

The perinatal outcome of FGR is dependent on the severity of growth restriction; an estimated fetal weight below the 3^rd^ centile and/or abnormal umbilical artery Doppler are strongly associated with adverse perinatal outcome [6]. A recent study has shown how the incidence of perinatal death is highest in those with a birth weight below the 2.3^rd^ centile, falling gradually with an increasing birth weight up to the 80^th^ and 90^th^ centiles, at which the lowest death rates occur [7].

FGR is a complex and multifactorial disorder affecting the fetal development that often results in multiple perinatal complications [8–10] and currently represents a major risk factors for long term poor neurological outcome. FGR is also associated with poor postnatal growth and numerous studies in both humans and animals have shown an association between low birth weight and development of cardiovascular disease including increased risk of hypertension, diabetes, dyslipidemia and coagulation in children and adults [9–16]. These observations were first made in 1989 by Barker and colleagues and confirmed in the last few decades [17, 18]. It has been postulated that cardiovascular remodelling is due to hemodynamic redistribution and adaptation to hypoxia and insufficient nutrition [17–22].

At present there is no effective treatment to reverse the course of FGR except delivery. Fetal growth restriction is probably among the obstetric entities where there is the greatest variation in clinical practice, in terms of monitoring and recommended gestational age at delivery. Prenatal recognition of FGR remains the main challenge in daily obstetric practice. Correct surveillance, antenatal management and timing of delivery can improve fetal and neonatal outcomes.

## Etiology

The causes of FGR are heterogeneous and can be classified as fetal, maternal, environmental, and placental. Small fetuses can be divided into two groups: non-placenta mediated growth restriction and placenta mediated [23].

The first group includes structural and chromosomal/genetic anomalies (trisomies 13 and 18; genetic conditions such as Russel Silver Syndrome), congenital infections (rubella, cytomegalovirus, toxoplasmosis) [24] and inborn errors of metabolism. The second and much more common group includes medical conditions that can affect placental function: pre-eclampsia, chronic hypertension and gestational hypertension are complicated by FGR in 30 to 40% of cases [23]; diabetes is complicated by FGR in 10 to 20% of cases irrespective of glycemic control [25]; maternal vascular disease, renal disease, thrombophilia, autoimmune disease, can lead to uteroplacental hypoperfusion thereby impairing fetal growth [26]; hypoxemia secondary to cardiac, respiratory and hematological disorders may also cause FGR.

Others maternal risk factors associated with an increased risk of a SGA neonate are maternal age ≥35 years and particulalry in women ≥40 years old [27], African American [28] or Indian/Asian ethnicity [29], nulliparity [30], social deprivation [31], body mass index (BMI) <20, BMI > 25 [32], alcohol intake [33], drug use (with cocaine use during pregnancy being the most significant) [34] and cigarette smoking [35]. Singleton pregnancies following IVF are also at increased risk for SGA [36]. Twin pregnancies have a high incidence of FGR: about 20–30% of dichorionic and 40% of monochorionic twin pregnancies will suffer from FGR [37].

## Definition and diagnosis

Multiple definitions of FGR have been suggested over the decades by National and Internationl Societies and experts (Table 1) [38, 39]. Despite this, there is currently no agreed upon diagnostic criteria for FGR. The American College of Obstetricians and Gynecologists (ACOG) defines FGR as an estimated fetal weight less than the 10^th^ centile [40]. The Royal College of Obstetricians and Gynaecologists (RCOG) uses fetal abdominal circumference (AC) or estimated fetal weight (EFW) <10^th^ centile to diagnose a FGR fetus [41]. Other Authors have suggested a cut off of the 3^rd^ centile to detect pregnancies at higher risk of adverse outcomes [42]. In 2002 the TRUFFLE group, which included 20 European Perinatal experts, defined fetal growth restriction as the combination of abdominal circumference <10^th^ centile and umbilical artery Doppler pulsatility index (PI) >95^th^ percentile [3]. Similarly, after prospectively assessing the adverse perinatal outcomes in over 1100 pregnancies where EFW at inclusion was <10^th^ centile, the PORTO group defined FGR as a combination of fetal smallness (EFW <5^th^ centile) and umbilical artery Doppler PI >95^th^ percentile [6]. More recently, early FGR has been defined by a consensus committe of international fetal medicine experts with solitary parameters ither EFW <3^rd^ centile, AC <3^rd^ centile or absent umbilical artery end diastolic flow [43].

**Table 1:** FGR definition in recent literature

Institution / Author	FGR definition
Baschat et al 2007 [101]	Combination of small fetal AC with elevated UA Doppler blood flow resistance
Cochrane 2013 [65]	Failure to reach the growth potential
DIGITAT 2012 [38]	EFW or AC <10th centile for gestational age
ACOG 2013 [40]	Fetuses with EFW <10th centile for gestational age
RCOG 2013 [41]	Small–for–gestational age (SGA) refers to an infant born with a birth weight less than the 10th centile. Fetal growth restriction (FGR) is not synonymous with SGA.
SOGC 2013 [39]	Intrauterine growth restriction refers to a fetus with a EFW <10th centile on ultrasound that, because of a pathologic process, has not attained its biologically determined growth potential.
PORTO 2013 [6]	EFW < 5th percentile & umbilical artery PI >95th percentile
TRUFFLE 2013 [3]	AC < 10th percentile & umbilical artery PI >95th percentile
Gordijin et al 2016 [43]	AC <3rd centile OR EFW <3rd centile OR AREDF OR Both of the following: 1) EFW or AC < 10th centile and 2) UtA PI >95th centile OR UA PI >95th centile.

*AC* abdominal circumference

*AREDF* absent/reversed umbilical artery end diastolic flow

*EFW* estimated fetal weight

*PI* pulsatility index

*UtA* uterine artery

*UA* umbilical artery

In its latest practice bulletin the ACOG acknowledges the fact that terminology for classifying fetuses and newborns who have failed to achieve normal weight is inconsistent [40]. Indeed, the acronyms SGA and FGR are often used interchangeably. However, fetuses with a weight <10 ^th^ percentile may be constitutionally small but healthy and not necessarily growth restricted. On the other hand, an estimated weight >10^th^ percentile does not necessarily denote normal fetal growth. Because of this, FGR shouldbe referred to fetuses with pathological smallness caused by an underlying functional problem and hence a definition including not only a biometric cut off but also Doppler indices of feto-placental function is currently agreed in most Fetal Medicine Units [3, 6, 43, 44].

FGR may also be classified by gestational age at onset as early and late, with an arbitrary cut-off conventionally set at 32 weeks: the extremes of the clinical spectrum of FGR differ not only for gestation at onset, but also for clinical manifestations, patterns of fetal deterioration, association with hypertensive disorders of the pregnancy and severity of placental dysfunction [45, 46]. Fetuses with late-onset disease do not present the same sequence of Doppler deterioration described for early-onset FGR [6]. Early-onset FGR represents 20–30% of all FGR and is associated with gestational hypertension and/or pre-eclampsia in up to 70%. On the other hand, late-onset FGR, which represents approximately 70–80% of cases of FGR, shows a weaker association with hypertensive disorders of the pregnancy, roughly 10% [6].

A prerequisite for a correct diagnosis of FGR is accurate dating of the pregnancy, most usually in the first trimester. The Hadlock formula is the most widely accepted method of estimating fetal weight using a composite sonographic measurement of fetal head, abdomen, and femur [47]. Fetal size is influenced by race, ethnicity, sex, parity, maternal size and genetic factors [48, 49]. In the 1990s, Gardosi et al. developed a method that used customized birth weights to identify the growth potential for individual fetuses: antenatal growth charts were customized for maternal characteristics including height, weight, ethnic origin and parity [50, 51]. The use of customized growth charts is purported to increase the antenatal detection of fetal growth restriction, improving the distinction between normal and abnormal growth, but whether this improves clinical outcomes still has to be demonstrated [51]. Customization is questioned by Intergrowth-21^st^ according to which growth patterns in healthy pregnancies are not considered to be modulated by ethnic and environmental conditions [52].

Ultrasound assessment of fetal anatomy and amniotic fluid volume is complementary to the Doppler investigation of fetoplacental circulation to distinguish FGR from constitutionally small fetuses and to identify the most likely underlying etiology of the fetal smallness (e.g., aneuploidy, viral infection, genetic syndromes). The role of determining the karyotype is very controversial; it may be offered in cases where there is a dissociation between growth, amniotic fluid and Doppler.

## Screening

Early screening to predict the likelihood of a FGR fetus include medical and obstetric history, uterine artery Doppler and maternal serum parameters [53]. Uteroplacental Doppler is the most powerful predictor of the clinical deterioration and the circumstances surrounding delivery [54]. The systematic review and meta-analysis conducted by Cnossen et al. in 2008 established uterine artery Doppler ultrasonography as a predictor of FGR, providing a more accurate prediction when performed in the second trimester than in the first-trimester [55]. Numerous studies have also shown that some maternal biochemical markers (e.g. pregnancy associasted plasma protein-a, PAPP-A; alfa-fetoprotein, AFP; human chorionic gonadotropin, hCG; Inhibin A) are associated with placental function and fetal growth, and their levels are altered in SGA and FGR pregnancies [56]. A low level first trimester PAPP-A should be considered a major risk factor for delivering a SGA neonate; the combination of uterine artery Doppler and maternal serum markers appears promising for improving prediction of SGA fetus, although predictive values are still poor [57–59]. Use of combination testing in the second trimester appears to predict adverse outcome related to placental insufficiency more effectively than first trimester screening [60].

## Role of fetal Doppler in FGR

The fetal vessels that are more commonly examined include umbilical artery, middle cerebral artery, and ductus venosus [61].

Early-and late-onset FGR epitomize two distinct clinical phenotypes of placental dysfunction and differ significantly in clinical progression. Early-onset FGR is associated with high impedance utero placental perfusion which in turn leads to elevated umbilical artery blood flow resistance once villous damage exceeds 30% [45].

The relationship between fetal size and growth and fetal Doppler indices in FGR is complex but in general Doppler deterioration is associated with absolute fetal size rather than growth velocity [62].

Late-onset FGR is more common but less severe with absent or mild placental abnormalities; umbilical artery Doppler may be normal, but fetuses may react with decreased middle cerebral artery (MCA) impedance in response to hypoxemia [63].

### Umbilical artery Doppler

Umbilical artery Doppler is the only measure that provides both diagnostic and prognostic information for the management of FGR [64]. A Cochrane systematic review reported that the use of umbilical artery Doppler was associated with a reduction in perinatal deaths, inductions of labor and cesarean deliveries [65]. Also according to RCOG the use of umbilical artery Doppler in a high-risk population has been shown to reduce perinatal morbidity and mortality, and should be the primary surveillance tool in the SGA fetus [41].

Umbilical artery flow identifies different degrees of impaired placental function. Absent or reversed end diastolic flow (AEDF or REDF) indicates an important reduction of blood flow and severe fetal deterioration.

Thanks to longitudinal studies of high-risk pregnancies, we know that the the transition from AEDF to REDF may be slow and gradual in early FGR. Absent end-diastolic velocities in the umbilical artery, if not associated with severe maternal disease, can last for days and weeks before abnormal heart rate pattern or delivery [66].

Reverse end-diastolic flow velocity represents an extreme abnormality in waveform and resistance, with a perinatal mortality of 50% and significant perinatal morbidity [67]. It has also been demonstrated that FGR fetuses with absent or reverse end-diastolic flow in the umbilical artery not only have an increased fetal and neonatal mortality but also a higher incidence of long-term permanent neurologic damage when compared with FGR fetuses with positive diastolic flow in the umbilical circulation [68].

### Middle cerebral artery Doppler

A condition of chronic hypoxia determines a fetal flow redistribution that manifests as vasodilatation in the brain circulation. Cerebral vasodilatation, easily detectable as a reduction in the PI of the middle cerebral artery (MCA) represents an adaptative mechanism in response to hypoxia.

Recently published data emphasize the association between abnormal MCA PI and adverse perinatal and neurological outcome [64]. MCA may be valuable for the identification of adverse outcome among late-onset FGR though its role in prediction is weak, independently of umbilical artery Doppler, which is often normal in these fetuses [69–71]. The cerebroplacental ratio (CPR) quantifies the redistribution of cardiac output by dividing the Doppler indices of the middle cerebral artery (MCA) with that of the umbilical artery. The PORTO study demonstrated the association between redistribution, either isolated or associated with umbilical artery PI >95^th^ centile, and adverse perinatal outcome [6, 63]. More recent data have shown significantly lower MCA PI and CPR among fetuses with EFW <10th centile diagnosed gestation beyond 32 weeks who experienced adverse perinatal outcomes in terms of intrapartum distress and abnormal cord pH [72]. Of note, such abnormal Doppler patterns have been related to histological signs of placental insufficiency [73]. It is estimated that in late-onset FGR fetuses abnormal CPR is present before delivery in 20 to 25% of cases [74]. Of note, it is important to underline that MCA Doppler is currently not included in any protocol for the diagnosis and the management of early FGR fetuses as insufficient data exists in prospective studies to define its role.

### Ductus venosus Doppler

Doppler examination of the ductus venosus (DV) plays an important role in the management of fetuses with early fetal FGR with the hope of improving the timing of delivery and outcome.

FGR is associated with increased ductus venosus (DV) shunting, and increasing impedance in the umbilical artery, has a graduated effect on the degree of shunting [20]. In contrast to alterations in umbilical artery and middle cerebral artery, which are early signs of adverse outcome, longitudinal studies have demonstrated that DV flow waveforms become abnormal only in advanced stages of fetal compromise [75–78].

It has been showed that the PI of the DV is related to pH at birth, with higher DV pulsatility associated with lower pH at birth [79].

In 2001 Hecher et al. described the time sequence of changes in fetal monitoring variables in fetal growth restriction; they found that ductus venosus PI and short-term variation of fetal heart rate are important indicators for the optimal timing of delivery before 32 weeks of gestation and correlate with fetal outcome at delivery [78]. Ferrazzi et al identified the temporal sequence of abnormal Doppler changes in the fetal circulation in early growth restricted fetuses. Early changes occurred in umbilical and middle cerebral arteries (AEDF and brain sparing respectively); late changes were significantly associated with perinatal death and included umbilical artery REDF and abnormalities in the DV Doppler (reverse A-wave in particular) [2]. Another study demonstrated that absent or reverse velocities in the DV during atrial contraction are associated with perinatal mortality independently of the gestational age at delivery [79]. Bilardo et al. showed that during the last 24 h before delivery DV pulsatility index for veins (PIV) and umbilical artery PI were significantly higher and STV lower in the adverse outcome group, while 2–7 days before delivery only DV PI was significantly higher. These results indicate that DV PI measurement is a good predictor of perinatal outcome and may be useful in determining the timing of the delivery in of early FGR fetuses [80]. In about 50% of cases, abnormal DV precedes the loss of short-term variability in computerized cardiotocography (CTG) [79], and in about 90% of cases it is abnormal 48 to 72 h before the biophysical profile (BPP) [80].

### Cardiac and aortic isthmus Doppler

Cardiac Doppler allows the evaluation of the functionality of the heart with the deterioration of FGR. Systolic and diastolic heart function, atrio-ventricular flows and ventricular outflows can be studied.

In 1988 a prospective longitudinal study by Rizzo et al. described the physiological patterns of blood flow velocity waveforms in normal and in SGA fetuses. In normal fetuses the ratio between the E velocity (early passive ventricular filling) and the A velocity (active ventricular filling during atrial contraction) increased progressively during pregnancy in both transmitral and transtricuspidal waveforms. In SGA fetuses, the E/A ratios did not increase during pregnancy and the values obtained were significantly lower than in normal fetuses [81]. In FGR fetuses the time to peak velocity at the level of the ascending aorta and pulmonary is also lower than in normal fetus, indicating an impairment of myocardial contractility [82].

The literature suggests a potential role for Doppler imaging of the aortic isthmus (AoI) in the clinical surveillance of fetuses with severe FGR. Retrograde flow in the AoI in growth-restricted fetuses correlates strongly with adverse perinatal outcome and neurological deficit in the infant [83, 84].

The myocardial performance index (MPI) is a more recently described parameter that may be useful in fetal monitoring. MPI, AoI PI, together with DV PIV, increase with progressive fetal deterioration. According to Cruz-Martinez et al., at the last examination before delivery the proportion of increased MPI (70.4%) was significantly higher than that of abnormal AoI PI (55.7%) and DV PIV (47.8%) [85]. A significantly higher MPI in growth restricted fetuses compared to appropriately grown fetuses was also demonstrated by Hassan et al, who additionally found a potential role of the MPI in the prediction of arterial and venous Doppler abnormalities in small for gestational age fetuses [86].

## Management

Despite being one of the most relevant and most commonly studied conditions in modern obstetrics, there has not been consensus among International Guidelines regarding the optimal management of early onset FGR in terms of monitoring and recommended gestational age at delivery, which can be due to the lack of comparability among studies and the paucity of randomized controlled trials available [3, 65, 87, 88]. Nevertheless, more recent data suggest that reliable protocols of surveillance and management are emerging now [3, 88].

When managing FGR fetuses clinicians focus on EFW, gestational age and fetal Doppler. A threshold of about 500 grams is often considered the value of EFW above which a fetus is potentially surviving outside the uterus, and which must be considered especially when evaluating the options of termination of pregnancy, invasive testing and delivery of a potentially viable fetus. Furthermore, an EFW <3^rd^ centile has been described as predictive of poor outcome [89].

Importantly, gestational age is the most significant determinant of both survival and intact survival [46]. A remarkable reduction in the gestational age cut off for neonatal survival has been achieved, and current neonatal practice has lead to the survival of fetuses born from 22 weeks onwards [90]. Recently published data report a better than expected prognosis for periviable small fetuses [91], however according to Visser et al. [92] active intervention by delivery of early FGR fetuses should not be recommended before 26 weeks as their outcome is comparable to that of AGA infants born at a 2-weeks earlier gestational age. Counseling plays a crucial role, however in a scenario of lacking evidence it is usually individualized on the basis of the clinical features and the option of pregnancy termination may be discussed when legally available.

Monitoring fetuses by using umbilical artery Doppler has been demonstrated to reduce the perinatal death rate [66, 69] though there are few clues as to when delivery should be undertaken. Abnormal umbilical artery PI is a feature of FGR according to the TRUFFLE group as a PI above the 95^th^ centile together with AC <10^th^ centile are the diagnostic criteria for FGR; a raised umbilical PI is suggestive of severe placental disease, which represents the most common cause for FGR [69]. However, as reported by Figueras et al. [64] the management based on umbilical artery alone cannot be effective in those cases of mild placental disease, which account for a proportion of the early onset FGR fetuses, which show reduced MCA pulsatility or cerebroplacental ratio (CPR) (or cerebro-umbilical C-U ratio). Unfortunately, there is not enough evidence to consider MCA Doppler effective in managing FGR fetuses. A recent review by DeVore et al [93] reports that an abnormal CPR is associated with adverse pre- and post-natal outcomes even in fetuses with early-onset SGA; however, it has never been demonstrated whether delivering earlier fetuses who show features of redistribution could add any benefit [64, 93, 94].

Ductus venosus (DV) Doppler is currently used in most European Specialist Perinatal Units as the reference for the management of FGR fetuses before 32 weeks. DV has been demonstrated to be the single strongest Doppler parameter to predict the short term risk of fetal death in early onset FGR [64] and there is good correlation between abnormal DV waveform and late stage acidemia. Absent or reversed A-wave have been reported to be associated with increased risk of intrauterine fetal death (40–70%) independently of the gestational age at delivery; DV PI >95th centile also confers higher risk of adverse outcome, although at lesser extent than that of reversed or absent A-wave [87]. According to Hecher et al. [76] DV is, together with computerized cardiotocography (cCTG), the parameter which last modifies before delivery, and the TRUFFLE group has demonstrated the benefits of a longer stay in utero, especially with regard to long term outcomes.

The biophysical profile has not been demonstrated to be beneficial in high risk pregnancies in terms of perinatal deaths and Apgar <7 at 5 min [33], it is not an accurate predictor of fetal acidemia [24, 34], and there is concern regarding the high false positive and false negative rate (up to 23% of instances of intrauterine fetal death in fetuses with BPP >6 and 11% in those with BPP >8) reported in early-onset very preterm FGR fetuses [46].

Conventional cardiotocography (CTG), along with the assessment of the baseline, the long term variability, accelerations and decelerations of the fetal heart rate, currently represents one of the main tools for the antenatal surveillance of the fetal wellbeing. CTG has been compared to no intervention in a Cochrane review of four randomized control trials. No improvement in perinatal mortality was shown [65, 95] and currently there is no other evidence supporting the use of conventional CTG in FGR fetuses [65, 89]. Short-term variability (STV) can be detected only using cCTG and becomes abnormal in the case of advanced fetal deterioration [77]. Current evidence suggests that cCTG is sensitive in the detection of advanced fetal deterioration providing information similar to DV reverse A-wave for the short-term prediction of fetal death.

Uterine artery Doppler may predict poor outcome in FGR but does not provide information sufficiently sophisticated to be considered effective in the management of the FGR fetuses [89, 96–98].

Which parameter is used to monitor FGR fetuses is not a more important issue than how often these fetuses should be assessed. A stage-based management protocol suggested by Figueras et al. [94], recommends fetal monitoring twice weekly up to 34 weeks if umbilical artery AEDF, every 24 to 48 h up to 30 weeks if reverse diastolic flow in the umbilical artery (REDV) or DV-PI > 95th centile, and every 12 to 24 h up to 26 weeks if spontaneous FHR decelerations, reduced STV (<3 ms) in the computerised cardiotocography, or reverse atrial flow in the DV.

There is no data as regards the decision for inpatient versus outpatient management of FGR fetuses. Most cases of isolated FGR are monitored in an outpatient setting even though the decision for inpatient monitoring can be taken on a subjective basis. Of note, 60–70% of cases of early FGR are associated with hypertensive complications of the pregnancy [3]. In such cases we believe that admission is advisable despite the lack of clinical data supporting this view.

## Timing of delivery

At present there is no effective intervention for FGR except delivery, and especially for early-onset FGR the timing is crucial and requires a balance between the risks of prematurity and the possibility of stillbirth and organ damage due to inadequate tissue perfusion [64, 90], unless severe maternal complications supervene [3, 88, 99].

Currently there is no consensus on what is the most appropriate trigger for delivery as the evaluation of the fetal status by Doppler indices and CTG cannot be assessed independently from the gestational age, which is the most significant determinant of both survival and intact survival, and fetal weight [90].

Though the GRIT study showed no clear benefit in delivering immediately or delaying delivery when a fetus is thought to be compromised, evidence from the TRUFFLE study shows how important it is to delay delivery in order to reduce the risk of cerebral palsy and poor neurodevelopmental outcome [79, 99] in this case based on deterioration in both the ductus venosus and STV from computerized CTG. However, this can be safely achieved only through protocols that integrate the best available evidence and reducing clinical practice variation [64].

Baschat et al. suggested the absent or reversed umbilical artery end diastolic velocity (AREDV) as the trigger for delivery, as it seems to have an independent impact on neurodevelopment from the late second trimester onward as representative of deepening hypoxemia [54], though this strategy was not tested in a prospective study.

The GRIT study was the first RCT which aimed to assess the timing for delivering FGR fetuses and concluded that “uncertainty” of the clinician as to whether deliver or not to deliver is related to the timing of delivery, which varied on average by only 4 days. Furthermore, fetuses with severe DV abnormalities at or beyond 28 weeks should be delivered after completion of steroids as there is evidence that reversed A-wave in the DV increases the risk of intrauterine fetal death at any gestational age [99].

According to the stage-based management protocol suggested by Figueras et al. [94] delivery by Caesarean section should be recommended at or after 34 weeks in case of umbilical artery AEDV, at or after 30 weeks if umbilical artery REDF or DV-PI > 95th centile and at or after 26 weeks if spontaneous fetal heart rate (FHR) decelerations, reduced short-term variability (<3 ms) in the cCTG, or reverse atrial flow in the DV Doppler.

The TRUFFLE is the only randomized controlled study which has evaluated a standardized monitoring and delivery protocol focussed on computerized CTG and DV Doppler. The “late” DV group (absent or reversed A-wave) was associated with significant improval in the rate of survival without impairment when compared to computerized CTG, and delivery was recommended in case of umbilical artery REDF between 30 and 32 weeks, umbilical artery AEDF between 32 and 34 weeks, or umbilical artery PI >95^th^ centile beyond 34 weeks. Safety nets for the computerised CTG were used in the TRUFFLE protocol, and may have contributed to the excellent outcomes in term of mortality and survival without impairment [3, 87].

Despite accumulating evidence suggesting that cerebral redistribution, defined by a reduction in the MCA PI below the 5^th^ centile or a reduction in the C-U ratio below the 2.5^th^ centile, may not be an entirely protective phenomenon, currently there is no evidence to recommend delivery before 34 weeks in fetuses who show features of cerebral redistribution [45, 64, 94].

Regarding the mode of delivery, 97% of women in TRUFFLE had Caesarean deliveries, compared with 98 and 85% in the cohorts reported by Baschat and GRIT, respectively [3, 87, 88, 100, 101]. It is of note that no studies have evaluated the optimal mode of delivery in early FGR fetuses.

## Outcomes

Early growth restricted fetuses by definition carry at least one of the major risk factors for perinatal morbidity and mortality, *i.e.* low birthweight and prematurity. Early and late-onset FGR are both associated with poor short- and long-term neurodevelopmental outcome, and also with cardiovascular and metabolic complications particularly in case of birth weigth <3^rd^ centile and gestation at delivery <26 weeks [64, 93]. Additionally, the cause of the growth restriction can impact on the short and long term prognosis, so especially for severely early growth restricted fetuses it is important to identify the cause by offering karyotyping and viral screen [32, 90]. This can allow to distinguish between fetuses who are affected by true FGR, and those who are constitutionally small (SGA) but with a guarded prognosis, particularly if the birthweight is below the 3^rd^ centile [102].

Few data exist regarding periviable growth restricted babies, a category which is commonly associated with poor outcome [92]. In 2014 Story et al [102] reviewed the outcomes of 20 FGR fetuses diagnosed <24 weeks and EFW <3^rd^ centile reporting a 67% preterm delivery rate and a better than expected survival to neonatal discharge rate of 60%. Such surprisingly good outcomes were confirmed by recently published data from a wider UK cohort of 245 cases of periviable FGR fetuses who showed an overall 41% neonatal survival rate and a 15% rate of delivery beyond 36 weeks, concluding that at a periviable gestational age both SGA and FGR can present in similar ways, and differentiation at the earliest possible time is crucial to allow appropriate management and counseling [91].

**Fig. 1:**
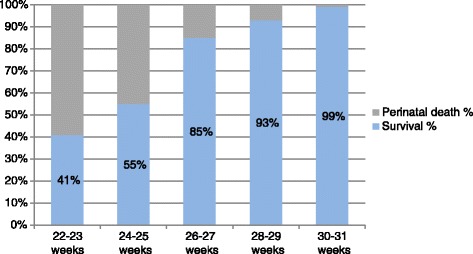
Short term survival across gestation at diagnosis

**Fig. 2:**
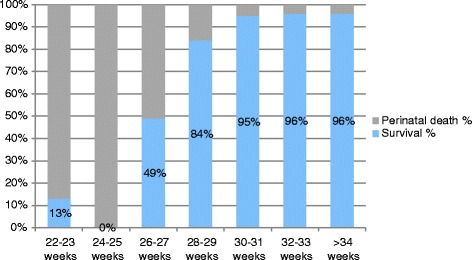
Short term survival across gestation at delivery

After 26 weeks the best results in terms of short term and 2-year outcomes were reported by the TRUFFLE group [3, 87], with 8% overall mortality (3% beyond 30 weeks), 24% severe neonatal morbidity, and low rates of bronchopulmonary dysplasia and poor neurodevelopmental outcome despite including fetuses who were on average 1 week younger and 300 g lighter if compared to the other RCT (GRIT) conducted on FGR fetuses [88]. Overall 69% of the 503 included fetuses survived without severe neonatal morbidity and these results were explained as a consequence of a more detailed and standardized protocol of surveillance. Allocation to delivery on the basis of absent/reversed A-wave in the DV accounted for a non significant increase in the stillbirth rate and a significant lower rate of abnormal neurodevelopmental outcome compared to the computerized CTG group, irrespective of the gestational age at inclusion [87]. According to Baschat et al. in-early onset FGR the nutritional and vascular restriction in placental function is limited with umbilical artery AREDV, and particularly reversed end-diastolic velocity is an independent risk factor for adverse motor and cognitive development [54]. Later in life no differences were reported in the rates of severe disability and individual domain scores between the two delivery arms in the GRIT study [86, 98] and results were comparable to other preterm cohorts without FGR [54]. Recently published data concerning short term survival of severe growth restricted fetuses across gestation at diagnosis and delivery are reported in Tables 2, 3 and in Figs. 1 and 2.

**Table 2:** Short term survival across gestation at diagnosis^a^

Gestation at diagnosis	Survival N	Perinatal death (IUD + NND) N	Survival %	Perinatal death (IUD + NND) %
22–23 weeks	49	69	41%	59%
24–25 weeks	52	42	55%	45%
26–27 weeks	126	22	85%	15%
28–29 weeks	188	15	93%	7%
30–31 weeks	164	2	99%	1%

Source: Data has been collated and fused from TRUFFLE 2013 [3] and Lawin O’Brien et al [91]

*IUD* intrauterine death

*NND* neonatal death

^a^Terminations of pregnancy (TOP) excluded

**Table 3:** Short term survival across gestation at delivery^a,b^

Gestation at delivery	Survival N	Perinatal death (IUD + NND) N	Survival %	Perinatal death (IUD + NND) %
22–23 weeks	1	7	13%	87%
24–25 weeks	0	30	0%	100%
26–27 weeks	51	52	49%	51%
28–29 weeks	141	27	84%	16%
30–31 weeks	173	10	95%	5%
32–33 weeks	111	5	96%	4%
>34 weeks	87	4	96%	4%

Source: Data has been collated and fused from TRUFFLE 2013 [3] and Lawin O’Brien et al [91]

*IUD* intrauterine death

*NND* neonatal death

^a^Lawin-O’Brien et al [91] 4 cases missing gestation at delivery

^b^Terminations of pregnancy (TOP) excluded

## Future directions

Delivery is acknowledged to be the only treatment for FGR at present and the most important studies have focused on how to monitor and when to deliver FGR fetuses in order to optimize the perinatal outcomes.

Currently there are no evidence-based therapies for early onset FGR but over the last decades it has been suggested that other approaches, namely nitric oxide (NO), Sildenafil and maternal plasma volume expansion may play a role in prolonging pregnancies and reducing Doppler deterioration in FGR fetuses [103–106].

Early FGR is associated with hypertensive disorders of the pregnancy and preeclampsia in up to 73 and 52% of the cases respectively [3]. It is hypothesized that the syndrome of pre-eclampsia stems from a failure of placental implantation and development [107] which leads to a failure in physiologic increase of uterine perfusion during pregnancy. Nevertheless, uteroplacental insufficiency is also a widely acknowledged cause of FGR. In 2001 Parra et al [103] demonstrated that in pregnancies complicated by FGR NO production is impaired if compared to normal and hypertensive cases, whereas more recent data suggest an increased NO production in FGR fetuses [108]. It is uncertain how and why NO pathways are abnormal in FGR fetuses and, most importantly, no data have supported a significant improvement in perinatal outcomes in mothers treated with NO. On the other hand, some evidences have shown that in pregnancies complicated by hypertension and growth restriction the combined therapy consisting in plasma volume expansion with enriched physiological solution and NO donors can improve maternal hemodinamics, prolong the pregnancy and increase the birthweight [106].

Animal models of fetal growth restriction have demonstrated that the phosphodiesterase 5 inhibitor Sildenafil citrate increases birth weight and improves uteroplacental blood flow and there is also little evidence from a study done on humans that Sildenafil increases birthweight in pregnancies complicated by ealy-onset preeclampsia [109]. STRIDER is the acronim of an ongoing prospective individual participant data study which will add clinical information as to whether Sildenafil can be safely and effectively used in human pregnancies [104].

Last, but not least, studies conduced on sheep have shown that local treatment at the uteoplacental site with the pro-angiogenetic factor VEGF-A improves fetal growth in growth restricted ewes [110, 111]. VEGF-A treatment was associated with a significant and long term (at least 4 weeks) increase in bloodflow and reduction in contractility of the uterine arteries in both pregnant and non pregnant animals [112]. These findings give hope that VEGF-A gene therapy can reverse the impaired uteroplacental perfusion which is associated with most cases of FGR even in humans, however further studies on both animal and human models are needed before any clinical application could be contemplated.

## Conclusions

In summary, a conservative management focused on the identification of etiology in periviable growth restricted fetuses is recommended, as prognosis can vary widely despite similar ultrasound findings at diagnosis. Beyond 26 weeks the current evidence suggests that a detailed surveillance protocol integrating fetal ductus venosus Doppler and computerized CTG allows better outcomes and delivery only when one or both become abnormal.

## Abbreviations

ACOG: American College of Obstetrician and Gynecologists
AEDF: Umbilical artery absent end diastolic flow
AFP: Alfa feto protein
AGA: Appropriate for gestational age
AoI: Aortic isthmus
AREDF: Umbilical absent or reverse end diastolic flow
BMI: Body mass index
BPP: Biophysical profile
CPR: Cerebro-placental ratio
CTG: Cardiotocography
C-U ratio: Cerebro-umbilical ratio
DV: Ductus venosus
EFW: Estimated fetal weight
FGR: Fetal growth restriction
FHR: Fetal heart rate
hCG: Human chorionic gonadotropin
MCA: Middle cerebral artery
MPI: Myocardial performance index
NO: Nitric oxide
PAPP-A: Pregnancy associated plasma protein-A
PI: Pulsatility index
RCOG: Royal College of Obstetricians and Gynecologists
RCT: Randomised controlled trial
REDF: Umbilical artery reverse end diastolic flow
SGA: Small for gestational age
STV: Short term variability
VEGF-A: Vascular-endotelial growth factor-A
